# Experience of a District General Hospital With a Diverse Community in Operated Colorectal Cancers According to Ethnic Background

**DOI:** 10.7759/cureus.36917

**Published:** 2023-03-30

**Authors:** Mahmoud Elnaggar, Ponnuthurai Pratheepan, Baskaran Paramagurunathan, Josie Colemeadow, Basim Hussein, Varvara Bashkirova, Kavya Pillai, Lucy Singh, Mehar Chawla

**Affiliations:** 1 Colorectal Surgery, North Middlesex University Hospital NHS Trust, London, GBR; 2 General Surgery, North Middlesex University Hospital NHS Trust, London, GBR

**Keywords:** stage, location, cancer, colorectal, ethnicity

## Abstract

Background

This study aimed to investigate disparities in colorectal cancer (CRC) patients’ demographics according to the five major ethnicities of patients living in the catchment area of North Middlesex Hospital.

Methodology

This retrospective study included CRC patients operated on between January 1, 2010, and December 31, 2014. Records dating to the end of the five-year follow-up were extracted anonymously from a database of CRC outcomes at the North Middlesex University Hospital NHS Trust. Comparisons were made according to ethnicity, patient demographics, type of presentation, cancer location, stage at diagnosis, recurrence, and mortality.

Results

A total of 176 adult patients were operated on for CRC between January 1, 2010, and December 31, 2014. The majority of the patients were referred as two-week wait target referrals. Emergency presentation of CRC was the highest in White non-UK patients. The White British Irish patients had their tumors mostly in the cecum, followed by the sigmoid colon, while the rectum followed by the sigmoid colon were the most common sites in the Black population. All study populations mainly presented with stage I disease, and the next highest incidence of cancers according to stage and ethnicity was stage IIIb in the Black population.

Conclusions

Differences in the ethnic background are important factors, especially in a diverse community, which can impact the age and mode of presentation of the disease, as well as the stage it starts to present. The location of the primary tumor, metastases, and recurrence sites are all affected by the ethnic background, which, subsequently, affect the survival of the patient.

## Introduction

Cancer of the colon is the world’s third most common cancer in males and the second most common cancer in females [1]. Colorectal cancer (CRC) will affect one in every 22 men and one in every 24 women in their lifetime [2]. Furthermore, 60% of cancers occur in developed countries [1]. Incidence rates are considerably higher in men than in women, with a sex ratio of 1.4:1. The incidence of CRC varies by more than a factor of 10 globally [3]. Australia and New Zealand have the highest incidence rates, with estimated age-standardized rates of 44.8 per 100,000 men and 32.2 per 100,000 women, with Europe and North America following closely. The lowest rates are seen in Africa at approximately 3.5 per 100,000 men and 3.0 per 100,000 women in Western Africa [3-5].

In the United Kingdom, access to medical care should not be affected by race or ethnic background. The Race Relations (Amendment) Act of 2000 emphasized the importance of ensuring racial equality of access to services [6].

Advanced age, male gender, a family background of colon or rectal cancer, a history of colorectal polyp, a history of inflammatory bowel disease, high body mass index (BMI), lack of physical activity, a history of type 2 diabetes, alcohol consumption, smoking, and a diet rich in red and processed meats are all linked with an increased risk of CRC [7-9]. However, increased calcium, fiber, folate, and vitamin D intake, as well as the use of non-steroidal anti-inflammatory drugs (NSAIDs) and hormone replacement therapy (HRT), may reduce the incidence of CRC [7-9]. Genetic makeup, epigenetics, diet, lifestyle, and the environment have all been found to be risk factors for this complex disease. CRCs having a genetic component, such as familial adenomatous polyposis and Lynch syndrome, account for 5% of cases, with another 25% clustering in families, suggesting weaker Mendelian inheritance [10,11]; nonetheless, the great majority of CRCs are sporadic.

Screening and surveillance guidelines for CRC include age and heritable components based on family history or an identified or suspected high-risk syndrome that changes the age and frequency of screening according to published guidelines. Previous guidelines did not include race as a factor, even though there is evidence that race engenders an increased risk for CRC [12,13]. In 2017, the Multi-society Task Force on Colorectal Cancer in the United States concluded that race is a contributing factor in CRC, a determinant that may lower the initial screening age [14].

According to data from the Surveillance, Epidemiology, and End Results (SEER) program, CRC incidence, stage at diagnosis, and death show considerable inequalities according to racial and ethnic groups in the United States, as an example of a diverse community such as the United Kingdom. Hispanics and Asians/Pacific Islanders had the lowest age-adjusted incidence and death, whereas African Americans had the highest age-adjusted incidence and death [15-17].

## Materials and methods

This was a retrospective study of CRC patients operated on between January 1, 2010, and December 31, 2014. Records dating to the end of the five-year follow-up were extracted anonymously from the database of CRC outcomes at the North Middlesex University Hospital NHS Trust, a District General Hospital (DGH) that serves a large catchment area in North London with a diverse community. The hospital’s catchment area covers parts of North London and East Hertfordshire, including the London Boroughs of Barnet, Enfield, and Haringey, as well as Broxbourne, East Hertfordshire, Epping Forest, and Uttlesford Districts. Comparisons were made according to ethnic background. The five main ethnic backgrounds seen in the electronic database of operated CRC cases during the five years of the study included White (British-Irish), other White (non-UK), Black (African and Afro-Caribbean), Asian, and other ethnic backgrounds (including Arabs).

Comparisons were made between these groups regarding sex differences, age at diagnosis of cancer, location of the tumor within the large bowel, cancer stage upon diagnosis, cases of recurrence after surgical treatment, and mortality within five years of diagnosis.

Data analysis

The data were analyzed using SPSS version 20.0 (IBM Corp., Armonk, NY, USA). Numbers and percentages were utilized to describe qualitative data. The significance of the acquired results was assessed at a 5% level. The tests that were employed included the chi-square test, Monte Carlo correction, and relative risk (RR).

## Results

A total of 176 adult patients were operated on for CRC between January 1, 2010, and December 31, 2014, in North Middlesex University Hospital NHS Trust according to electronic CRC records provided anonymously by the informatics department.

The highest number of patients belonged to the White ethnicity; however, the total number of British and Irish Whites (42 patients) ranked third following White non-UK nationals (66 patients) and Black patients (44 patients). There were 10 Asian patients while 14 patients belonged to different ethnicities including Arab backgrounds (Table 1).

**Table 1:** Distribution of the studied cases according to different parameters in ethnicity (n = 176).

	N	%
Ethnicity background		
White British Irish	42	23.9
Other white non-UK	66	37.5
Black	44	25.0
Asian	10	5.7
Others	14	8.0
Age at diagnosis in decades		
40s	13	7.4
50s	35	19.9
60s	34	19.3
70s	48	27.3
80s	46	26.1
Location of cancer at diagnosis		
Appendix	4	2.3
Cecum	47	26.7
Ascending colon	9	5.1
Hepatic flexure	4	2.3
Transverse colon	3	1.7
Splenic flexure	6	3.4
Descending colon	6	3.4
Sigmoid colon	51	29.0
Rectum	46	26.1
Stage of cancer at diagnosis		
I	65	36.9
IIa	19	10.8
IIb	18	10.2
IIc	16	9.1
IIIa	17	9.7
IIIb	18	10.2
IIIc	13	7.4
IVa	4	2.3
IVb	3	1.7
IVc	3	1.7

**Figure 1:**
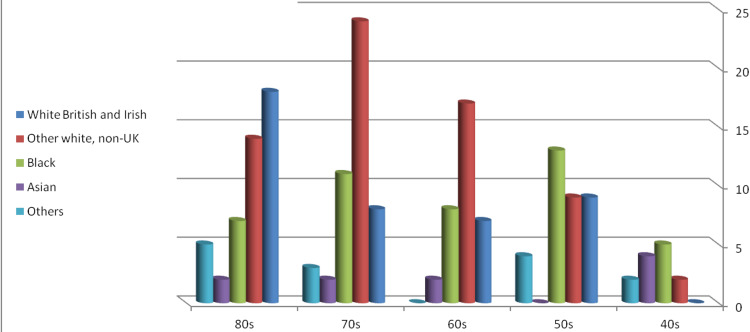
Age at the time of cancer diagnosis.

Most White British Irish patients were in their 80s, while other White non-UK ethnic background patients were in their 70s. Black patients were mostly in their 50s, whereas Asians showed the highest age distribution of patients in their 40s. Patients belonging to other ethnic backgrounds were mostly in their 80s (Figure 1).

There was a statistically significant finding that the White British Irish population developed cancers at a later age than Black patients with an RR of 1.905 (confidence interval (CI) = 0.999 to 3.664, p < 0.05) (Tables 2, 3).

**Table 2:** Comparison between the different studied groups of ethnicity according to age at diagnosis in decades. *: Statistically significant at p ≤ 0.05. χ^2^: Chi-square test; MC: Monte Carlo

Age at diagnosis in decades	White British Irish (n = 42)		Other White, non-UK (n = 66)		Black (n = 44)		Asian (n = 10)		Others (n = 14)		χ^2^	^MC^p
	N	%	N	%	N	%	N	%	N	%		
40s	0	0.0	2	3.0	5	11.4	4	40.0	2	14.3	17.461^*^	<0.001^*^
50s	9	21.4	9	13.6	13	29.5	0	0.0	4	28.6	7.259	0.109
60s	7	16.7	17	25.8	8	18.2	2	20.0	0	0.0	5.466	0.225
70s	8	19.0	24	36.4	11	25.0	2	20.0	3	21.4	4.445	0.351
80s	18	42.9	14	21.2	7	15.9	2	20.0	5	35.7	9.710^*^	0.038^*^
χ^2 ^(^MC^p)	36.047^* ^(0.001^*^)											

**Table 3:** Comparison between the Black and White British Irish ethnicities regarding age at diagnosis. Relative risk is 1.905 and the confidence interval is 0.990 to 3.664 for the theory that White British Irish patients developed cancers at a later age than Black patients.

	Ethnicity	
	Black (n = 44)	White British Irish (n = 42)
Age at diagnosis in decades		
50s	13	9
80s	7	18

The majority of White patients were females, whether belonging to the British Irish background (24 females and 18 males) or other non-UK whites (36 females and 30 males), while the opposite was noted with the Black ethnicity (25 males and 19 females). The Asian population showed equal sex distribution (five males and five females). Finally, nine males and five females did not belong to the above-mentioned ethnic backgrounds and were considered to belong to other ethnic backgrounds, including Arabs.

**Figure 2:**
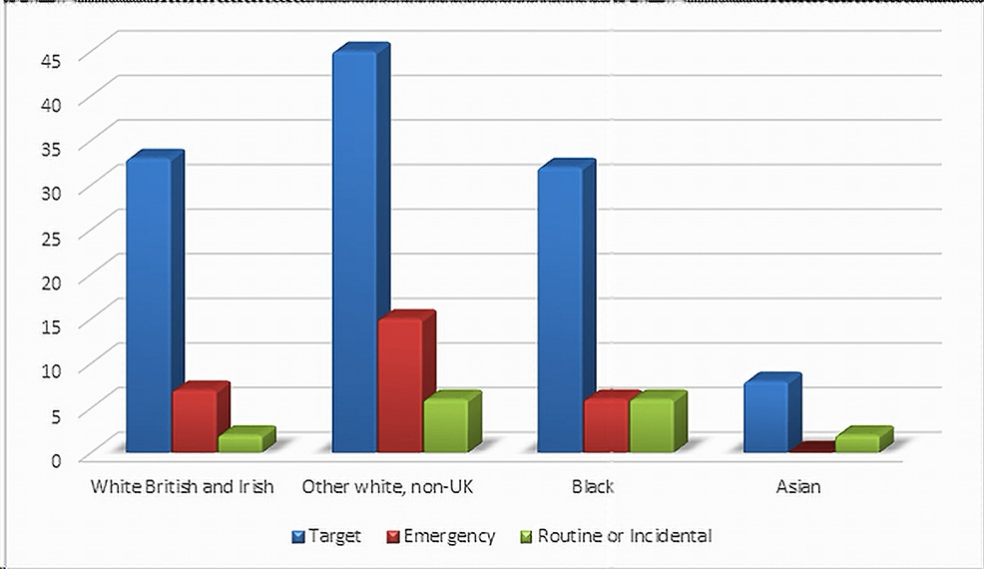
Type of referral to the specialist service.

According to the type of initial referral, most patients from each group were referred as two-week wait target referrals: 78.5% of the White British Irish, 68% of the White non-UK group, 72% of the Black group, 80% of the Asian group, 42.8% of the other backgrounds group. Emergency presentation of CRC was the highest in the White non-UK population, with 22.7% of the patients presenting for the first time with CRC as an emergency presentation (e.g., emergency rectal bleeding, bowel obstruction, or abdominal pain). The White British Irish group showed 16.6% of emergency presentations, the Black population showed 13.6% of emergency presentations, and the Asian population showed no emergency presentations (Figure 2).

**Figure 3:**
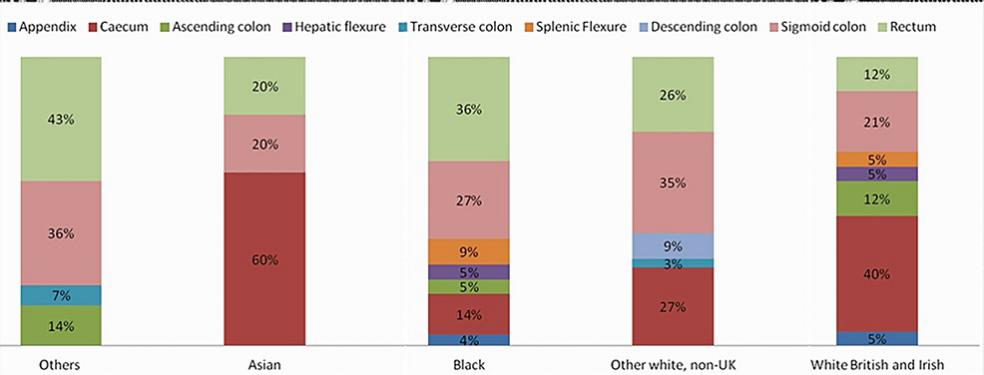
Location of the primary colorectal tumor.

Regarding tumor location, the White British Irish population had their tumors mostly in the cecum, followed by the sigmoid colon, which was the same as the Asian population. However, the rectum followed by the sigmoid colon were the most common sites in the Black population. The White non-UK group had their cancers mostly in the sigmoid followed by the cecum (Figure 3).

Rectosigmoid cancers (rectum + sigmoid colon) were more common in White non-UK and Black ethnicities than the White British Irish, whereas cecal cancers were more common in the White British Irish compared to White non-UK and Black patients (RR = 1.637, CI = 1.090 to 2.457, p < 0.05) (Tables 4, 5).

**Table 4:** Comparison between the different studied groups of ethnicity according to the location of cancer at diagnosis *: Statistically significant at p ≤ 0.05. χ2: Chi-square test; MC: Monte Carlo

Location of cancer at diagnosis	White British Irish (n = 42)		Other White, non-UK (n = 66)		Black (n = 44)		Asian (n = 10)		Others (n = 14)		χ^2^	^MC^p
	N	%	N	%	N	%	N	%	N	%		
Appendix	2	4.8	0	0.0	2	4.5	0	0.0	0	0.0	4.113	0.330
Cecum	17	40.5	18	27.3	6	13.6	6	60.0	0	0.0	18.887^*^	0.001^*^
Ascending colon	5	11.9	0	0.0	2	4.5	0	0.0	2	14.3	10.284^*^	0.015^*^
Hepatic flexure	2	4.8	0	0.0	2	4.5	0	0.0	0	0.0	4.113	0.334
Transverse colon	0	0.0	2	3.0	0	0.0	0	0.0	1	7.1	4.307	0.299
Splenic flexure	2	4.8	0	0.0	4	9.1	0	0.0	0	0.0	6.381	0.109
Descending colon	0	0.0	6	9.1	0	0.0	0	0.0	0	0.0	6.885	0.077
Sigmoid colon	9	21.4	23	34.8	12	27.3	2	20.0	5	35.7	2.966	0.571
Rectum	5	11.9	17	25.8	16	36.4	2	20.0	6	42.9	9.224^*^	0.049^*^
χ^2 ^(^MC^p)	57.578^*^ (<0.001^*^)											

**Table 5:** Comparison between the White British Irish group and the other White non-UK and Black groups regarding the location of cancer at diagnosis. Relative risk is 1.637 and the confidence interval is 1.090 to 2.457 for the theory that rectosigmoid cancers (rectum + sigmoid colon) are more common in White non-UK and Black ethnicities than White British Irish ethnicity, while cecum cancers are more common in White British Irish compared to White non-UK and Black patients.

	Ethnicity	
	White British Irish (n = 42)	Other White, non-UK + Black (n = 110)
Location of cancer at diagnosis		
Cecum	17	24
Rectosigmoid cancers (sigmoid colon + rectum)	14	68

Relative risk was 1.637 with a CI of 1.090 to 2.457 for the theory that rectosigmoid cancers (rectum + sigmoid colon) are more common in White non-UK and Black ethnicities than the White British Irish ethnicity, whereas cecum cancers are more common in the White British Irish compared to White non-UK and Black patients.

**Figure 4:**
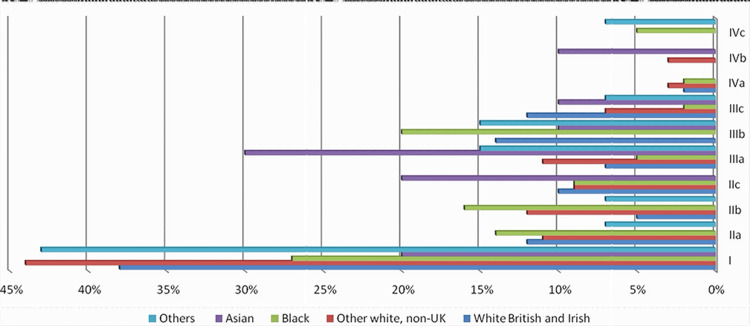
Tumor stage at diagnosis.

The cancer stage at diagnosis showed that all study populations mainly presented with stage I disease. The next highest incidence of cancers according to stage and ethnicity was stage IIIb in the Black population (Figure 4).

There were six recurrences of cancer out of 42 patients operated on in the White British Irish population, of which four were in the liver. Five recurrences were documented for the other White population, of which two were lung recurrences. There were eight recurrences in the Black population, of which four were in the lungs. The Asian population showed one recurrence which was in retroperitoneal lymph nodes. There was no specific distribution of the three recurrences noted in the other ethnicity population.

**Figure 5:**
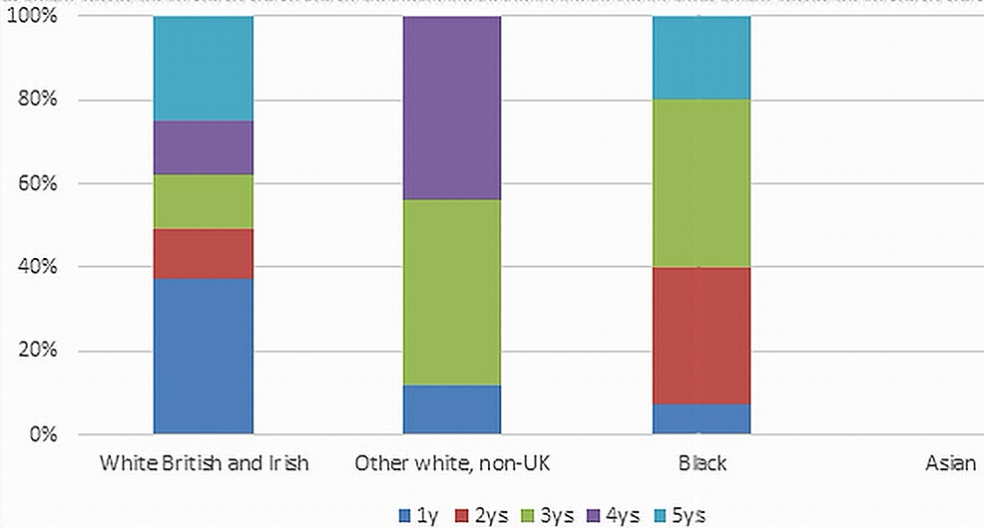
Mortality percentages by ethnicity.

There were 14 (33.3%) mortalities within the first five years following diagnosis in the White British Irish population, while there were 16 (24.2%) mortalities during the same follow-up period in the White non-UK group. The highest mortality of 15 (34%) patients within five years of diagnosis was noted in the Black population. There were no mortalities in the Asian group of patients (Figure 5).

The site of recurrence of cancer was studied as well in relation to both primary tumor and ethnic background, and the findings are shown in Table 6.

**Table 6:** Site of recurrence of cancer in relation to both primary tumor and ethnic background.

Ethnicity	Primary tumor	Recurrence site
White British Irish	Transverse colon	Liver
	Sigmoid	Liver, lung
	Cecum	Peritoneal
	Ascending colon	Liver
	Rectum	Liver
Other white, non-UK	Cecum	Umbilicus
	Rectum	Lung
	Descending colon	Liver, lung
	Cecum	Transverse colon (metachronous)
	Rectum	Local recurrence
Black	Sigmoid	Lung
	Rectum	Adrenals, liver
	Rectum	Local recurrence
	Rectum	Lung
	Rectum	Liver
	Rectum	Local recurrence and uterus
	Rectum	Lung
	Appendix	Lung
Asian	Sigmoid	Retroperitoneal lymph nodes
Others	Ascending colon	Lung
	Transverse colon	Retroperitoneal lymph nodes

## Discussion

While discussing the ethnic or racial background of CRCs, one has to bear in mind the difference between countries where similar studies were conducted discussing similar care topics. For example, in the United States, the key determinants of CRC screening include socioeconomic characteristics and physician supply. According to Soneji et al., adjusting for factors can explain the discrepancies between Black and White people regarding screening [18].

On comparing the incidence of CRC in the White population in the United States between 1992-1996 and 2010-2014, it was discovered that the rates increased from 7.5 to 11.0 per 100,000, whereas the incidence rate in the Black population increased from 11.7 to 12.7 per 100,000. Rectal cancer rates increased faster in Whites (from 2.7/100,000 to 4.5/100,000) than in Blacks (from 3.4/100,000 to 4.0/100,000) from 2010 to 2014. Rectal cancer rates were similar among Blacks and Whites [19]. In this study, rectosigmoid cancers (rectum + sigmoid colon) were more common in White non-UK and Black ethnicities than in White British Irish, whereas cecal cancers were more common in White British Irish compared to White non-UK and Black patients (RR = 1.637, CI = 1.090 to 2.457, p < 0.05) (Table 4).

According to a recent systematic review [20], CRC is frequent in the White population in the United Kingdom, although it is uncommon among UK-based South Asians. In this study, only 10 Asian patients were operated on for CRC during the study period, which is about 5.6% of the total cancer operation load of the hospital. However, we did not study cancer incidence in the community per ethnicity as this study focused mainly on operated cancers from the time of diagnosis.

A major prospective study conducted in the United States found that differences in the prevalence of known/suspected risk variables did not fully explain ethnic disparities in the age-adjusted incidence of CRC. Japanese Americans (both sexes) and African American women had a higher risk of CRC than Whites after accounting for differences in risk variable distribution [21].

In a heterogeneous, contemporary, population-based sample of 877,662 cancer patients in the United States, Ellis et al. [22] discovered continuing differences in survival for breast, prostate, lung, and colorectal cancer across racial/ethnic groups. Cancer-specific mortality was 36% higher in Black men (hazard ratio (HR) = 1.36, 95% CI = 1.30 to 1.43) and 34% higher in Black women in patients with CRC (HR = 1.34, 95% CI = 1.28 to 1.41). In this study, the five-year mortality of Black patients was 34%, with the highest mortalities occurring in the second year following cancer surgery. The five-year mortality of White non-UK patients was 38%, with the highest mortalities occurring in the third year of operation. Finally, in the White British Irish patients, the five-year mortality was 33%, with most deaths occurring in year one postoperatively (Figure 5).

A retrospective, single-center investigation from Israel comprised 401 individuals with pathologically confirmed CRCs diagnosed between 2008 and 2015 [23]. These were split into two groups: Jewish (n = 334) and Arab (n = 67). The tumor stage, location, histologic grade, and mortality rate were collected and compared retrospectively between the two groups. Results showed that Arabs were significantly younger at diagnosis, which is comparable to the findings of this study in that there was an evident age discrepancy at the time of diagnosis between different ethnic groups. The White British Irish population had their cancers mainly in their ninth decade followed by the sixth decade with almost equal distribution between the seventh and eighth-decade cancers (Figure 1). Figure 1 also shows that other White non-UK patients had their cancers during their eighth, seventh, ninth, and sixth decades, implying that White populations tend to have CRCs later in life. However, in the Black population, CRCs occurred in the sixth, eighth, seventh, and ninth decades, implying that their CRCs presented earlier. There was a statistically significant finding that the White British Irish population developed cancers at a later age than Black patients with an RR of 1.905 (CI = 0.999 to 3.664, p < 0.05) (Table 2).

Going back to the Israeli study, the tumor distribution through the colon was comparable between both groups and was characterized by a distal predominance. In the current study, the other ethnicity groups, which included Arabs, showed a similar outcome of distal tumors being more common than right colonic tumors. It appears that right-sided cancers are the most common in White and Asian populations. Furthermore, Black patients and other ethnicity groups had left-sided cancers more commonly than right-side cancers (Table 3). Arabs had a much greater rate of the advanced stage upon diagnosis, according to the Israeli study, when compared to Jews, which adds evidence to the findings of this study regarding differences in presenting a stage of cancer based on ethnicity. As shown in Figure 4, White British Irish patients had their cancers at diagnosis mostly in stage I, followed by stage IIIb, while Black patients had stage I as the most common presenting stage, followed by stage IIb. Similarly, other White non-UK patients had stage I as the most common presentation. However, stages IIa and IIIa equally shared second place. Of note, Asian patients had 50% of the presentations as stage III.

In the study from Israel, the mortality rates of both groups were comparable. In this study, mortalities in the other ethnicity group reached 50% within five years of operation compared to White populations (33% and 38%) for the two White populations studied here (Figure 5).

The increased prevalence of anal sexual practices among younger adults in Western countries, according to a study by Habel et al., may also be linked to the discrepancies in CRC frequencies among different ethnicities [24]. Anal intercourse may result in more contact between the anus and the rectum than vaginal intercourse due to lower condom use [25]. Sexually transmitted infections may have a role in CRC due to the close proximity of the rectum to the anus and the known carcinogenic association of human papillomavirus with anal cancer [26]. As human papillomavirus has a physiologic relationship with CRC, high-risk sexual behaviors among young adults can be a contributing factor [27].

Finally, it is well-recognized that inflammatory bowel disease and other causes of intestinal irritation increase the risk of CRC in White individuals. The mortality incidence rate for African Americans was 40.7%, which is the highest among races in the United States. Furthermore, Blacks have a CRC distribution that favors metastatic illness when compared to non-Hispanic Whites (NHWs). Black patients had a lower five-year survival rate than White patients, and their tumors had a higher proportion of *Kirsten rat sarcoma* (*KRAS*) gene mutations, making CRC more aggressive [28]. According to this study, the disparity in CRC in the United States, particularly between Blacks and NHWs, is likely multifactorial. It is uncertain if ensuring equal access to health care will be enough to overcome some of the biological factors that make CRC more aggressive in African Americans. In this study, Black patients presented with a high incidence of stage III and IV tumors (Figure 4), had a younger age at diagnosis (Figure 1), and had a high mortality rate of 34% within five years, which was the second following the other ethnicity group which showed 50% mortality but with a lower total number of patients (14 patients compared to 44 Black patients).

Tapan et al. [29] reported that overall survival was significantly lower in Black patients than in White patients, with a median overall survival of 1.9 and 2.5 years, respectively. Black race was found to be a significant risk factor for death in a multivariate analysis (HR = 1.7, CI = 1.01-2.9, p = 0.0467). Despite the assumption of equal access to health care and socioeconomic status within a safety-net hospital system, the findings of this study are consistent with previous research on CRC survival in Black patients and emphasize the importance of examining other risk factors such as genetic and pathogenic differences.

Furthermore, the type of initial referral to specialist service in this study was mostly the target type of referral in all ethnicity subtypes, while emergency referral was the second most common in White and Black populations with a significant increase in percentage in the Black population of about 22%.

The recurrence site was studied per population showing that the most common recurrence site in the White British Irish population was the liver, with 66.6% of recurrences, while 40% of the recurrences in the White non-UK patients were in the lungs. In Black patients, 50% of recurrences were in the lungs.

The limitations of this study include factors that may be related to ethnicity but are not genetically determined, such as socioeconomic status, ethnicity-related behaviors, or medical conditions, which can also predispose individuals to cancers as an indirect effect of ethnic background, thus likely impacting the outcome.

Further prospective studies should be conducted to confirm the effect of ethnicity on cancer progression and outcome.

## Conclusions

CRCs are a major health challenge, and investigating their risk factors and pathogenic differences is a crucial aspect of management as it can modify the screening strategies and help tailor investigations and allocate resources accurately.

Differences in ethnic background are important factors, especially in a diverse community, which can impact the age and mode of presentation of the disease, as well as the stage it starts to present with. The location of the primary tumor, metastases, and recurrence sites are all affected by the ethnic background, which, subsequently, affects the survival of the patient.
